# Study on the Ultrasonic-Assisted Extraction Process of Anthocyanin from Purple Cabbage with Deep Eutectic Solvent

**DOI:** 10.3390/molecules30061281

**Published:** 2025-03-13

**Authors:** Lifen Meng, Pengpeng Ding, Ye Tan, Yinying Zhang, Jun Zhao

**Affiliations:** 1 School of Chemical Engineering, Guizhou University of Engineering Science, Bijie 551700, China; mlfsandy@163.com (L.M.); haizhiwu664@126.com (P.D.); zhangyu852022@163.com (Y.T.); tuyijiao@163.com (Y.Z.); 2 Analysis and Testing Center, Guizhou University of Engineering Science, Bijie 551700, China

**Keywords:** anthocyanin, purple cabbage, deep eutectic solvent, ultrasonic-assisted

## Abstract

In this paper, purple cabbage was used as raw material for ultrasonic-assisted extraction of anthocyanin with deep eutectic solvent. The effects of extraction solvent type, solid–liquid ratio, moisture, extraction temperature, and time on the yield of anthocyanin from purple cabbage were investigated by single factor test, and the feasibility of this extraction method was verified by standard addition recovery test. The test results showed that the optimal extraction results could be obtained when DES-5 (choline chloride/1, 2-propylene glycol/water) is used as extraction solvent, with solid–liquid ratio of 1:32, moisture of 50%, extraction temperature of 50 °C, and extraction time of 80 min. Under these conditions, the yield of anthocyanin extract purple cabbage reached 21.6%, and the recovery rates were 85.62–87.75%. Therefore, DES was a promising environmentally friendly solvent for extracting anthocyanins instead of organic solvent extraction.

## 1. Introduction

Among the all-natural pigments contained in plants, anthocyanin may be the most recognized for its biological activity [1]. At present, how to obtain anthocyanin from plants that are beneficial to the human body is the focus of the public’s attention [2]. So far, solvent extraction has been one of the most commonly used methods [3]. This method is less picky about equipment but consumes a large amount of time and energy. Meanwhile, the high temperature required makes it easy to lose the effective constituents [4]. Microwave-assisted extraction can significantly improve the reaction efficiency and reduce energy consumption, but it will also cause certain harm to human health [5]. Compared with the two methods, ultrasonic-assisted extraction has a much lower cost and higher efficiency [6]. However, traditional organic solvents such as methanol and ethanol are often applied as extraction solvents, which are, in fact, flammable, explosive, volatile, and unfriendly to the environment and human health [7]. Therefore, a new green and efficient method for extraction of anthocyanin from purple cabbage is needed [8].

Among vegetables known for high concentrations of anthocyanin, purple cabbage is one of the most commonly used sources of these pigments in food production and is regarded as a suitable raw material for extracting natural dyes [9]. As a natural anthocyanin food colorant, purple cabbage anthocyanin has irreplaceable nutritional value and is highly appreciated all over the world. This extraordinary and nutritious vegetable is an excellent source of health-promoting ingredients that can help strengthen our immune system [10].

**Figure 1:**
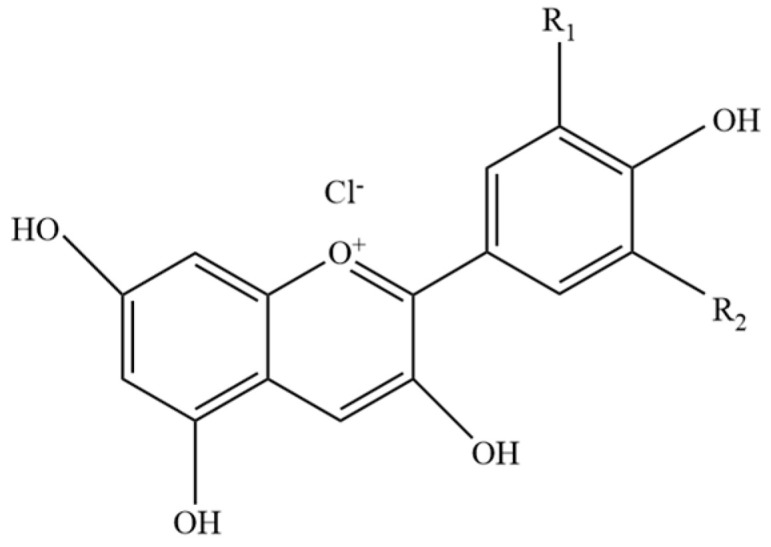
Basic structure of anthocyanin.

Anthocyanin has a structure composed of two aromatic rings (Figure 1), which are connected by three carbons in an oxygen heterocyclic ring (i.e., the chromium ring with the second aromatic ring in position 2). Anthocyanin is composed of two or three chemical units, including glycosyl or flavane ring (anthocyanin), sugars, and possible acylated groups [11]. The traditional solvent extraction method is the most commonly used for extracting anthocyanins, phlorotannins, lignin, triterpene saponins, curcumins, etc. [12]. Pusty K et al. [13] studied the effect of process parameters on the extraction of phytochemicals from red cabbage by the application of ultrasonication and temperature.

The concept of DES was proposed by Abbott et al. in 2003 [14]. For the past few years, a new green extraction technology, deep eutectic solvent (DES), has emerged [15]. Generally, DES is composed of hydrogen bond acceptors (quaternary ammonium salts and choline chloride) and hydrogen bond donors (such as amino acids, carboxylic acids, and sugars, etc.), which form a stable crystal structure under certain conditions [16]. This crystal structure gives DES properties similar to ionic liquids, characterized by inter-molecular interactions, especially hydrogen bonds [17]. As a result, DES is featured with excellent physical and chemical properties, such as negligible volatility at room temperature, non-flammability, and adjustable viscosity [18]. DES is easy to prepare and highly soluble in different kinds of compounds because it can form hydrogen bonds. Compared with common solvents, DES is cheap, safe, low in toxicity, non-volatile, thermostable, nonflammable, sustainable, and biodegradable. For its safety and environmental protection characteristics, DES plays a vital role in green technology [19].

The solvent selected for the study was deep eutectic solvent (DES) prepared by choline chloride and citric acid. The ultrasound-assisted extraction process was modeled using an adaptive neuro-fuzzy inference system (ANFIS) algorithm and integrated with the genetic algorithm for optimization purposes. Corrale et al. [20] used ultrasonic-assisted extraction technology to study the process conditions for extracting anthocyanin from grapes. The results showed that such technology was more suitable for the extraction of anthocyanins, and the extraction yield was more than 50% higher than that of the conventional solvent extraction methods.

The microwave-assisted extraction method utilizes the energy of the microwave to initiate molecular motion so that the solvent and the sample are rapidly heated, the water in the sample cells is vaporized, and the cells are expanded and ruptured, releasing anthocyanins and other effective substances. Zou et al. [21,22] applied this method to extract mulberry anthocyanin and obtained the optimal extraction conditions. Using acidified methanol of 59.6%, anthocyanin of 54.72 mg was extracted from the mulberry powder of 1.0 g, with a solid–liquid ratio of 1:23, at the power of 425 W, with an extraction time of 132 s.

In this paper, according to the properties of anthocyanin in purple cabbage and the extraction process, with the appropriate DES, the effects of extraction process parameters such as solid–liquid ratio, moisture, extraction temperature, and extraction time on the yield of anthocyanin were studied by single factor test to optimize the extraction process parameters, and the extracted anthocyanin content was detected using UV-vis.

## 2. Results and Discussion

### 2.1. Results of Infrared Spectrum Characterization of DES

**Figure 2:**
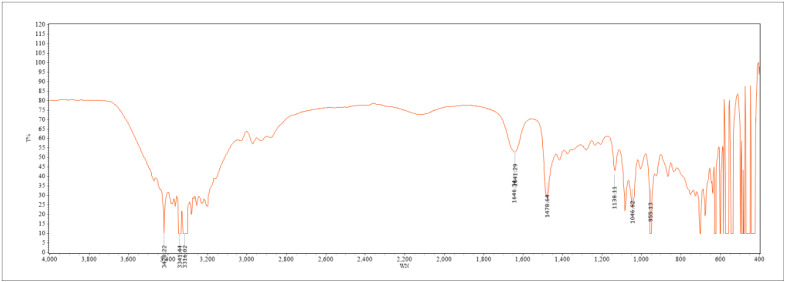
Infrared spectrum of DES.

The structure and main functional groups of DES (choline chloride/1,2-propylene glycol/water) were characterized by Fourier transform infrared spectroscopy. It can be seen from Figure 2 that the O-H group stretched and vibrated in the interval of 3500–3200 cm^−1^, showing a wide band characteristic, which mainly resulted from the formation of intermolecular hydrogen bonds. The characteristic peak of choline chloride −CH_3_ appeared at about 1478 cm^−1^. Due to the stretching and vibration of secondary alcohol’s C-O bond, two significant peaks were generated at about 1138 cm^−1^ and 1046 cm^−1^. The peak at about 1646 cm^−1^ was caused by the in-plane scissor bending vibration of the O-H bond. Due to the high moisture of the solvent, some solute peaks may be covered. The above results indicated that DES was associated with hydrogen bonds without chemical changes [23].

### 2.2. Results of Single Factor Test

#### 2.2.1. Effect of Extraction Solvent Type on the Yield of Anthocyanin

**Figure 3:**
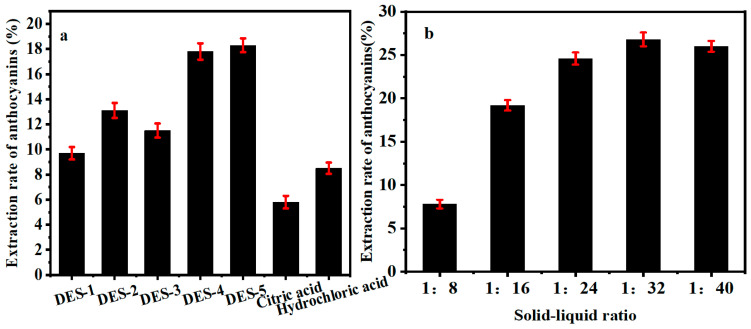
(**a**) Effect of extraction solvents on the extraction rate of anthocyanins, (**b**) Effect of solid–liquid ratios on the extraction rate of anthocyanins.

As shown in Figure 3a, among the seven types of solvents, DES-5 (choline chloride/1,2-propylene glycol/water) was the most effective extraction solvent, followed by DES-4 (choline chloride/glycerol/water), DES-2 (choline chloride/lactic acid/water), DES-3 (choline chloride/glucose/water), DES-1 (choline chloride/citric acid/water), hydrochloric acid solution, and then citric acid solution of 4%. Among the five kinds of DES, DES-5 (choline chloride/1,2-propylene glycol/water) extracted purple cabbage anthocyanin of the highest yield, that is, 18.1%, while DES-1 (choline chloride/citric acid/water) ranked last, only reaching 9.5%. Compared with the conventional solvents, a citric acid solution of 4% obtained an anthocyanin yield of 6.5%, and a hydrochloric acid solution of pH_3_ 9.3%. The yield of anthocyanin extracted by DES-5 (choline chloride/1,2-propylene glycol/water) was 8.8% higher. The higher yield of anthocyanin in DESs was likely benefited by the physical and chemical properties of DES that were more conducive to the dissolution of anthocyanin. Therefore, DES-5 is determined as the extraction solvent of purple cabbage anthocyanin.

#### 2.2.2. Effect of Solid–Liquid Ratio on the Yield of Anthocyanin

It can be seen from Figure 3b that the yield of anthocyanin increased with the increase of solid–liquid ratio. When the solid–liquid ratio reached 1:32, the anthocyanin yield reached the maximum, and as the solid–liquid ratio continued to increase, the anthocyanin yield began to decrease. The reason may be that the larger amount of extraction solvent resulted in the higher dispersion of purple cabbage powder in the solvent, creating a wider contact area with the solvent, which was conducive to the extraction of anthocyanin. When the solid–liquid ratio was greater than 1:32, the yield decreased. It is possible that when the solid–liquid ratio reached 1:32, most of the anthocyanin had been dissolved, and the following addition of extraction solvent diluted the solution instead, causing the decrease of anthocyanin yield. In addition, anthocyanin was relatively stable at high concentrations, and the stability would decrease as the concentration decreased. The absorbance may also be reduced due to the decomposition of anthocyanin. Therefore, the perfect solid–liquid ratio for anthocyanin extraction is determined to be 1:32.

#### 2.2.3. Effect of Water Content on the Yield of Anthocyanin

**Figure 4:**
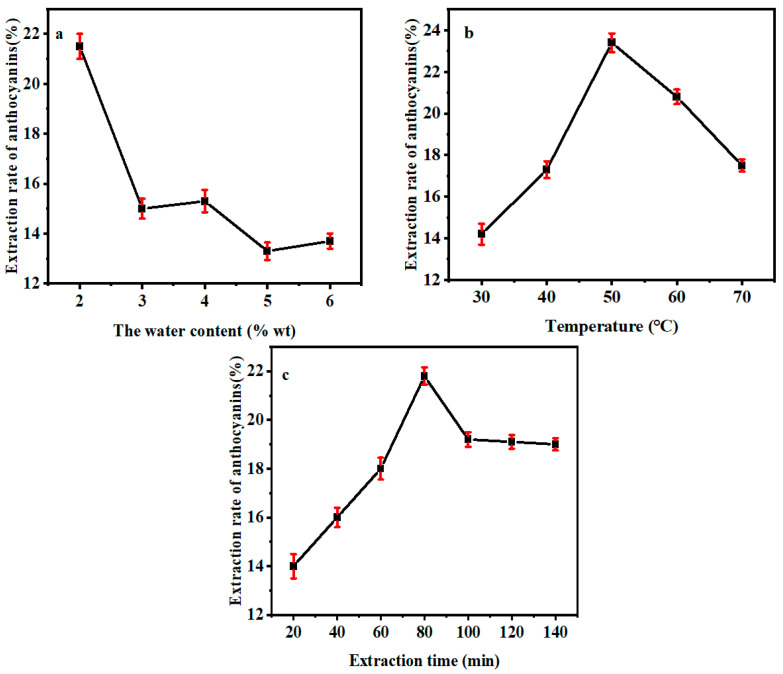
(**a**) Effect of water content on the extraction rate of anthocyanins, (**b**) Effect of temperature on the extraction rate of anthocyanins, and (**c**) Effect of time on the extraction rate of anthocyanins.

As shown in Figure 4a, with the increase of % wt, the yield of anthocyanin decreased gradually. When 2% wt of water was added, the maximum yield of anthocyanin was obtained. When the water content was more than 2% wt, the yield of anthocyanin decreased. The reason may be that the variation of water content would change the viscosity of DES. Excessive water destroyed the hydrogen bond acting force between the extraction solvent and anthocyanin, weakening their interaction and thus reducing the yield of anthocyanin. Therefore, the optimum water content for the subsequent experiment of anthocyanin extraction is determined to be 2% wt [24,25].

#### 2.2.4. Effect of Extraction Temperature on the Yield of Anthocyanin

From Figure 4b, it can be seen that the yield of purple cabbage anthocyanin increased with the rise of extraction temperature. When the temperature ascended to 50 °C, the yield of anthocyanin reached the peak. As the temperature rise continued, the yield of anthocyanin began to descend. The reason may be that the increase in temperature accelerated the dissolution rate of purple cabbage powder in a solvent. At the same time, the heat would also change the structure of the cell membrane, leading to a faster dissolution of anthocyanin into the extraction solvent. However, due to the thermal sensitivity of anthocyanin, excessive temperature will lead to the degradation of anthocyanin and the decrease of anthocyanin yield. Therefore, the optimal extraction temperature for the subsequent experiment of extracting anthocyanin is determined to be 50 °C.

#### 2.2.5. Effect of Extraction Time on the Yield of Anthocyanin

From Figure 4c, it can be learned that the yield of anthocyanin increased with the extension of extraction time. When the extraction went on for 80 min, the yield of anthocyanin reached the peak. As the time prolonged, the amount of extracted anthocyanin decreased. It is probable that the anthocyanin kept dissolving during the extraction and almost emptied in about 80 min. However, due to the long extraction time, the anthocyanin in the extraction solvent was oxidized and degraded, resulting in the reduction of anthocyanin yield. Therefore, the optimum time for extracting purple cabbage anthocyanin is determined to be 80 min.

### 2.3. Results of Standard Addition Recovery Test

According to literature reports, the detection methods for anthocyanins generally included UV-visible spectrometry [26], infrared spectrometry (IR) [27], and high-performance liquid chromatography (HPLC) [28,29,30].

In order to verify the accuracy of the determination methods in this experiment, under the optimal conditions, a recovery rate test was performed by using purple cabbage as a sample and selecting three standard addition levels, that is, 0.8 mg/mL, 1.0 mg/mL, and 1.5 mg/mL. According to the calculation, the anthocyanin recovery rates were 86.87%, 85.62%, and 87.75%, respectively, as shown in Table 1. The results showed that the determination methods of this experiment are feasible [31,32].

## 3. Experimental Section

### 3.1. Materials and Instruments

In this experiment, purple cabbage (Latin name *Brassica oleracea* var. capitata rubra) was purchased from the local market. Anthocyanin standard substance (95% UV) was purchased from Chengdu Desite Biotechnology Co., Ltd. (Chengdu, China). Choline chloride (ChCl), Hydrochloric acid, Vanillin, Methanol, Citric acid, Lactic acid, Glycerol, Glycerol, and 1,2-Propylene glycol were all purity analyses. Pulverizer (Wenling Big Machinery Co., Ltd., Wenling, China), Electronic analytical balance (Shimadzu, San Jose, CA, USA), Ultraviolet-visible spectrophotometer (Shimadzu), Fourier transform infrared spectrometer (WQF-530A) from Beijing Beifen-Ruili Analytical Instrument (Group) Co., Ltd. (Beijing, China), DF-101Z Collective constant temperature heating magnetic stirrer (Shanghai Yushen Instrument Co., Ltd., Shanghai, China), and TDZ4-WS Table-type low-speed auto-balancing centrifuge (Changsha Maijiasen Instrument Equipment Co., Ltd., Changsha, China) were used for this experiment.

### 3.2. Methods

#### 3.2.1. Sample Treatment

Fresh purple cabbage was selected, washed with water, dried to constant weight at 60 °C, and then crushed to obtain purple cabbage powder, which was sealed and stored.

#### 3.2.2. Preparation of Extraction Solvent

A variety of DESs were prepared. The preparation methods are shown in Table 2. In a water bath of 60 °C, the magnetic stirring was carried out for 4–6 h to obtain a crystal clear liquid, which was cooled to room temperature for later use.

#### 3.2.3. Preparation of Conventional Solvents

Accurately weigh 4.00 g of citric acid, dissolve it in distilled water, then transfer it to a 100 mL volumetric flask and make up to volume. Finally, obtain a citric acid solution with a mass fraction of 4% and seal it for future use. Prepare pH 3.0 buffer solution and seal it for future use.

### 3.3. Extraction Methods

#### 3.3.1. Extraction Processes

**Figure 5:**
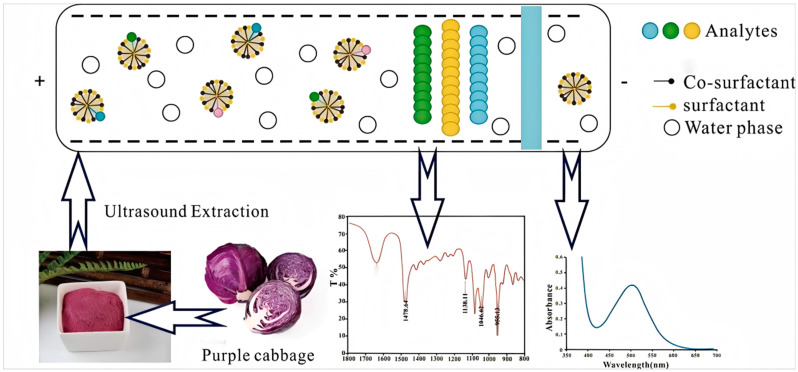
Extraction process diagram of anthocyanins from purple cabbage.

Mixed a certain amount of purple cabbage powder, co-surfactants, and DES in a certain proportion and stir evenly. After ultrasonic extraction, obtain the supernatant by centrifugation. Take out the supernatant and measured the absorbance by UV-vis, record the data, and calculate the anthocyanin concentration and anthocyanin yield. This was a method that had never been reported in the literature, and using DES reagent to extract anthocyanins was a novel approach (Figure 5 Extraction process).

#### 3.3.2. Extraction of Anthocyanin

The dried purple cabbage powder of 1.00 g was accurately weighed and put into the extraction container. DES was then added. After evenly stirring the mixture, ultrasonic extraction was started under certain conditions. The extracted liquid was centrifuged for 15 min at a speed of 4000 r/min to remove the waste residue. The supernatant left was the DES crude extract.

#### 3.3.3. Extraction of Anthocyanin by Conventional Solvents

Purple cabbage powder of 1.00 g was accurately weighed and put into the extraction container. The extracting solution was then added as per the solid–liquid ratio of 1:10 (g/mL). At 4000 r/min, the extracted mixture was centrifuged for 15 min to remove the waste residue. The supernatant left was the crude extract of conventional solvents.

#### 3.3.4. Calculation of Anthocyanin Production

The aforesaid extracting solution of 1 mL was added with methanol for constant volume in a volumetric flask of 25 mL, and then the liquid of 1 mL was taken to replace the standard substance solution. According to the method, the color analysis was performed. The absorbance of the sample solution was measured. Using the regression equation, the anthocyanin concentration (mg/mL) and yield of the sample solution were calculated. The formula is as follows

(1) $$w=\frac{C\times V\times n}{m}\times 100\%,$$

where C—the anthocyanin concentration in the sample solution obtained from the anthocyanin standard curve, mg/mL, V—volume of sample solution, mL, n—dilution multiple of sample solution, m—amount of purple cabbage powder (g), and w—anthocyanin content.

### 3.4. Determination of Anthocyanin Content in Actual Samples

The standard solution of 1 mL with a concentration of 0.8–1.5 mg/mL were added to the DES containing 1.00 g of sample substance, respectively. Ultrasonic-assisted extraction was then performed under the DES-5 as extractant, the solid–liquid ratio was 1:32, the solvent moisture was 2% wt, the extraction temperature was 50 °C, and the extracting time was 80 min. The extracted mixture was centrifuged for 15 min at 4000 r/min. The supernatant of 1 mL was put into a volumetric flask of 25 mL for constant volume, and then the solution of 1 mL was placed in a colorimetric tube of 25 mL. Vanillin–methanol solution and concentrated hydrochloric acid solution were then added for photophobic reaction at 30 °C for 30 min. After measuring the absorbance, the anthocyanin concentration was calculated by the regression equation.

## 4. Conclusions

In this paper, an ultrasonic-assisted extraction method with DES was established to extract anthocyanin from purple cabbage. This study fully shows that the DES of choline chloride/1,2-propylene glycol/water works perfectly well on the extraction of purple cabbage anthocyanin, and its effect can be greatly improved by the ultrasonic-assisted extraction method. Compared with the reported methods, this method provides a green and effective approach for the extraction of anthocyanin from purple cabbage, opening up a new way for the extraction of other bioactive substances.

## Figures and Tables

**Table 1:** Standard addition recovery rates of anthocyanin.

Samples	Measured of Sample (mg/mL)	Standard Addition (mg/mL)	Measured of Standard Addition Sample (mg/mL)	Recovery Rate %
Purple cabbage	0.0351	0.8	0.73	86.87
		1.0	0.89	85.62
		1.5	1.35	87.75

**Table 2:** Preparation methods for five kinds of DES.

DES	Composition			Mass Ratio
	Hydrogen Bond Acceptor	Hydrogen Bond Donor	Water	
DES-1	ChCl	Citric acid	Water	1:1:4
DES-2	-	Lactic acid	-	1:1:4
DES-3	-	Glucose	-	1:1:4
DES-4	-	Glycerol	-	1:1:4
DES-5	-	1,2-Propylene glycol	-	1:1:4

## Data Availability

Data are contained within the article and Supplementary Materials.

## Supplementary Materials

The following supporting information can be downloaded at: https://www.mdpi.com/article/10.3390/molecules30061281/s1, Figure S1: UV spectrum of anthocyanins; Figure S2: UV spectrum of DES; Figure S3: Standard curve of anthocyanin.

## References

[1] Giampieri F., Cianciosi D., Alvarez-Suarez J.M., Quiles J.L., Forbes-Hernández T.Y., Navarro-Hortal M.D., Machì M., Casanova R.D.J.P., Espinosa J.C.M., Chen X. (2023). Anthocyanins: What Do We Know until Now?. J. Berry Res..

[2] Romera-Fernández M., Berrueta L.A., Garmón-Lobato S., Gallo B., Vicente F., Moreda J.M. (2012). Feasibility Study of FT-MIR Spectroscopy and PLS-R for the Fast Determination of Anthocyanins in Wine. Talanta.

[3] Noypitak S., Jaitrong N., Terdwongworakul A. (2022). Detection of Spongy Pulp in Guava Using Light Properties and Near Infrared Spectroscopy. Int. Food Res. J..

[4] Nogales-Bueno J., Baca-Bocanegra B., Jara-Palacios M.J., Hernández-Hierro J.M., Heredia F.J. (2017). Evaluation of the Influence of White Grape Seed Extracts as Copigment Sources on the Anthocyanin Extraction from Grape Skins Previously Classified by near Infrared Hyperspectral Tools. Food Chem..

[5] Stuppner S., Mayr S., Beganovic A., Beć K., Grabska J., Aufschnaiter U., Groeneveld M., Rainer M., Jakschitz T., Bonn G.K. (2020). Near-Infrared Spectroscopy as a Rapid Screening Method for the Determination of Total Anthocyanin Content in Sambucus Fructus. Sensors.

[6] Li H.D., Liang Y.Z., Cao D.S., Xu Q.S. (2012). Model-Population Analysis and Its Applications in Chemical and Biological Modeling. TrAC Trends Anal. Chem..

[7] Mansour F.R., Bedair A., Hamed M., Magdy G., Ali I., Locatelli M. (2024). Applications of (Natural) Deep Eutectic Solvents in Liquid Phase Microextraction: A Review. Microchem. J..

[8] Cheng J., Zheng C., Xu K., Zhu Y., Song Y., Jing C. (2024). Sequential Separation of Critical Metals from Lithium-Ion Batteries Based on Deep Eutectic Solvent and Electrodeposition. J. Hazard. Mater..

[9] Dong W., Yang X., Zhang N., Chen P., Sun J.H., Harnly J.M., Zhang M. (2024). Study of UV-Vis Molar Absorptivity Variation and Quantitation of Anthocyanins Using Molar Relative Response Factor. Food Chem..

[10] Elhamarnah Y., Qiblawey H., Nasser M. (2024). A Review on Deep Eutectic Solvents as the Emerging Class of Green Solvents for Membrane Fabrication and Separations. J. Mol. Liq..

[11] Lamarca R.S., Ferreira S.D.S., Paganini E.R., Ferreira N.D.S., Ayala-Durán S.C., Isquibola G., de Lima Gomes P.C.F., Amaral C.D.B., Magnani M., Franco D.F. (2024). Sustainable Cadmium Extraction from Sewage Sludge Samples: A Novel Approach with Hydrophobic Deep Eutectic Solvents and Ultrasound-Assisted Extraction (HDES-UAE) Prior to ICP-MS Analysis. J. Mol. Liq..

[12] Yang S., Wang C., Li X., Wu C., Liu C., Xue Z., Kou X. (2021). Investigation on the Biological Activity of Anthocyanins and Polyphenols in Blueberry. J. Food Sci..

[13] Pusty K., Dash K.K., Giri S., Bhagya Raj G.V.S., Tiwari A., Shaikh A.M., Béla K. (2024). Ultrasound Assisted Phytochemical Extraction of Red Cabbage by Using Deep Eutectic Solvent: Modelling Using ANFIS and Optimization by Genetic Algorithms. Ultrason. Sonochem..

[14] Abbott A., Capper G., Davies D., Rasheed R.K., Tambyrajah V. (2003). Novel Solvent Properties of Choline Chloride/Urea Mixtures. Chem. Commun..

[15] Zhang H., Tsao R. (2016). Dietary Polyphenols, Oxidative Stress and Antioxidant and Anti-Inflammatory Effects. Curr. Opin. Food Sci..

[16] Petreska Stanoeva J., Damjanovski V., Cichna-Markl M., Stefova M. (2023). Anthocyanin Fingerprinting as an Authentication Testing Tool for Blueberry, Aronia, and Pomegranate Juices. Eur. Food Res. Technol..

[17] Dai F., Shi J., Yang C., Li Y., Zhao Y., Liu Z., An T., Li X., Yan P., Dong C. (2023). Detection of Anthocyanin Content in Fresh Zijuan Tea Leaves Based on Hyperspectral Imaging. Food Control..

[18] Fujiki H., Tobase K., Muguruma H. (2024). Electrochemical Determination of the Procyanidins in Peanut Skin Using a Carbon Nanotube Electrode. Anal. Sci. Int. J. Jpn. Soc. Anal. Chem..

[19] Li Z., Liu Q., Lu X., Chen X., Zhao J. (2024). Deep Eutectic Solvents with Low Viscosity for the Determination of Polycyclic Aromatic Hydrocarbons in Environmental Water Samples by Dispersive Liquid-Liquid Microextraction. Microchem. J..

[20] Corrales M., Toepfl S., Butz P., Knorr D., Tauscher B. (2008). Extraction of Anthocyanins from Grape By-Products Assisted by Ultrasonics, High Hydrostatic Pressure or Pulsed Electric Fields: A Comparison. Innov. Food Sci. Emerg. Technol..

[21] Zou T., Wang D., Guo H., Zhu Y., Luo X., Liu F., Ling W. (2012). Optimization of Microwave-Assisted Extraction of Anthocyanins from Mulberry and Identification of Anthocyanins in Extract Using HPLC-ESI-MS. J. Food Sci..

[22] Mondal S., Syed U.T., Pinto E., Leonardo I.C., Romero P., Gaspar F.B., Barreto Crespo M.T., Sebastian V., Crespo J.G., Brazinha C. (2024). Sustainable Production of Nanoemulsions by Membrane-Assisted Nanoemulsification Using Novel Aroma-Based Hydrophobic Deep Eutectic Solvents for Enhanced Antifungal Activities. J. Clean. Prod..

[23] Yang D., Wang Y., Peng J., Xun C., Yang Y. (2019). A Green Deep Eutectic Solvents Microextraction Coupled with Acid-Base Induction for Extraction of Trace Phenolic Compounds in Large Volume Water Samples. Ecotoxicol. Environ. Saf..

[24] Petrochenko A.A., Orlova A., Frolova N., Serebryakov E.B., Soboleva A., Flisyuk E.V., Frolov A., Shikov A.N. (2023). Natural Deep Eutectic Solvents for the Extraction of Triterpene Saponins from *Aralia elata* var. *mandshurica* (Rupr. & Maxim.). J. Wen. Mol..

[25] Velásquez P., Bustos D., Montenegro G., Giordano A. (2021). Ultrasound-Assisted Extraction of Anthocyanins Using Natural Deep Eutectic Solvents and Their Incorporation in Edible Films. Molecules.

[26] Zendehdel A.A., Sorouraddin S.M., Farajzadeh M.A. (2024). Development of Salt-Induced Homogeneous Liquid-Liquid Extraction Using a Deep Eutectic Solvent Performed in a Narrow-Bore Tube for the Extraction of Zn(II), Cu(II), and Cd(II) Ions from Honey Samples. Anal. Methods Adv. Methods Appl..

[27] Shishov A.Y., Bulatov A.V. (2024). Automated Microextraction Separation of Lead from Vegetable Oils for Determination by Atomic Absorption Spectrometry. J. Anal. Chem..

[28] Zhang Y., Hunter J.R., Ullah A., Shao Q., Shi J. (2024). Lignin Derived Hydrophobic Deep Eutectic Solvents for the Extraction of Nanoplastics from Water. J. Hazard. Mater..

[29] Aktaş H., Kurek M.A. (2024). Deep Eutectic Solvents for the Extraction of Polyphenols from Food Plants. Food Chem..

[30] Huang J., Guo X., Xu T., Fan L., Zhou X., Wu S. (2019). Ionic Deep Eutectic Solvents for the Extraction and Separation of Natural Products. J. Chromatogr. A Incl. Electrophor. Other Sep. Methods.

[31] Foroutani Z., Afshar Mogaddam M.R., Ghasempour Z., Ghareaghajlou N. (2024). Application of Deep Eutectic Solvents in the Extraction of Anthocyanins: Stability, Bioavailability, and Antioxidant Property. Trends Food Sci. Technol..

[32] Prakash S., Goswami A., Patil R., Mitra A., Kutty N.N. (2024). An Eco-Friendly Approach to Extract Anthocyanins from Rose Flowers Using Natural Deep Eutectic Solvents. Ind. Crops Prod..

