# Supplementary material for: Study on the Ultrasonic-Assisted Extraction Process of Anthocyanin from Purple Cabbage with Deep Eutectic Solvent
Source: Molecules. 2025 Mar 13;30(6):1281. doi: 10.3390/molecules30061281 (PMC11944879; doi:10.3390/molecules30061281)
Supplement: Supplementary file 1 [file molecules-30-01281-s001.zip › molecules-3291005-supplementary.pdf]

1. Determination of maximum absorption wavelength

The standard anthocyanin of 1ml was diluted with methanol and then transferred to a volumetric flask of 10ml for constant volume. Vanillin-methanol mixture was then added, and concentrated hydrochloric acid solution later. After 30 min of photophobic reaction, UV-vis spectrophotometer was used for scanning within the range of 200~700 nm to determine the maximum absorption wavelength of anthocyanin.

2. Ultraviolet spectrogram of anthocyanin

As shown in Figure S1, the anthocyanin standard substance had the maximum absorption wavelength at 500 nm, so the absorbance measured at 500 nm was taken as the standard in this experiment. Figure S2 shown the UV spectrum of DES, which had no absorption peak at 500nm, so it could not interfere with the detection of anthocyanins.

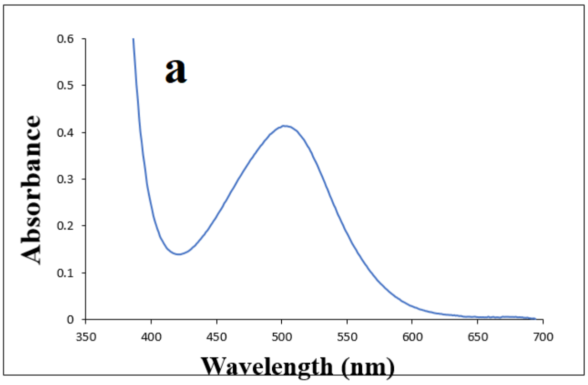

Figure S1. UV spectrum of anthocyanins

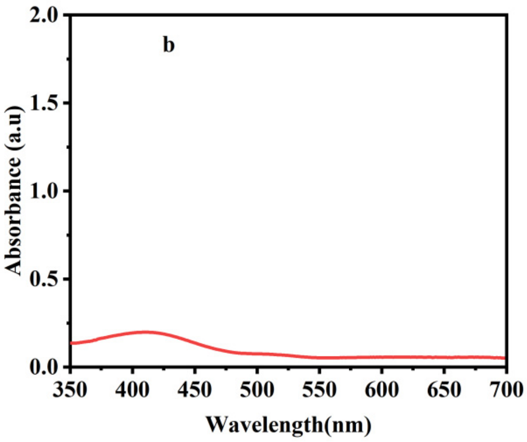

Figure S2. UV spectrum of DES

3. Standard curve of anthocyanin

It can be seen from Figure S3 that the curve has an excellent linear relation in the concentration range of 0.48 mg/ml~1.12 mg/ml, and the regression equation is, y=0.3126x+0.0709 (R^2=0.99725). The LOD and LOQ of this method are 0.1 mg/ml and 0.85 mg/ml, respectively. The recovery rates of this method are above 85%.

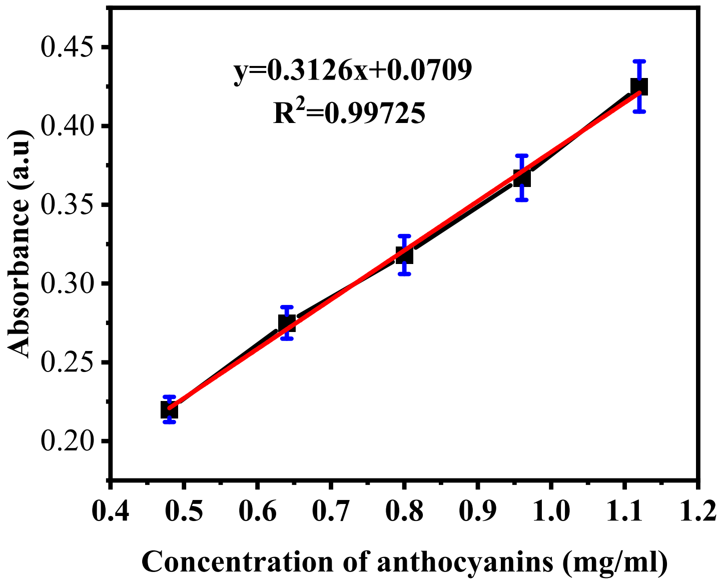

Figure S3. Standard curve of anthocyanin
